# Spatial Memory and Taxis-Driven Pattern Formation in Model Ecosystems

**DOI:** 10.1007/s11538-019-00626-9

**Published:** 2019-06-04

**Authors:** Jonathan R. Potts, Mark A. Lewis

**Affiliations:** 1 School of Mathematics and Statistics, University of Sheffield, Hicks Building, Hounsfield Road, Sheffield, S3 7RH UK; 2 Department of Mathematical and Statistical Sciences, CAB632, University of Alberta, Edmonton, AB T6G 2G1 Canada; 3 Department of Biological Sciences, CAB632, University of Alberta, Edmonton, AB T6G 2G1 Canada

**Keywords:** Advection–diffusion, Animal movement, Chaos, Movement ecology, Population dynamics, Taxis

## Abstract

Mathematical models of spatial population dynamics typically focus on the interplay between dispersal events and birth/death processes. However, for many animal communities, significant arrangement in space can occur on shorter timescales, where births and deaths are negligible. This phenomenon is particularly prevalent in populations of larger, vertebrate animals who often reproduce only once per year or less. To understand spatial arrangements of animal communities on such timescales, we use a class of diffusion–taxis equations for modelling inter-population movement responses between $N \ge 2$ populations. These systems of equations incorporate the effect on animal movement of both the current presence of other populations and the memory of past presence encoded either in the environment or in the minds of animals. We give general criteria for the spontaneous formation of both stationary and oscillatory patterns, via linear pattern formation analysis. For $N=2$, we classify completely the pattern formation properties using a combination of linear analysis and nonlinear energy functionals. In this case, the only patterns that can occur asymptotically in time are stationary. However, for $N \ge 3$, oscillatory patterns can occur asymptotically, giving rise to a sequence of period-doubling bifurcations leading to patterns with no obvious regularity, a hallmark of chaos. Our study highlights the importance of understanding between-population animal movement for understanding spatial species distributions, something that is typically ignored in species distribution modelling, and so develops a new paradigm for spatial population dynamics.

**Electronic supplementary material:**

The online version of this article (10.1007/s11538-019-00626-9) contains supplementary material, which is available to authorized users.

## Introduction

Mathematical modelling of spatial population dynamics has a long history of uncovering the mechanisms behind a variety of observed patterns, from predator–prey interactions (Pascual 1993; Lugo and McKane 2008; Sun et al. 2012) to biological invasions (Petrovskii et al. 2002; Hastings et al. 2005; Lewis et al. 2016) to inter-species competition (Hastings 1980; Durrett and Levin 1994; Girardin and Nadin 2015). These models typically start with a mathematical description of the birth and death processes and then add spatial aspects in the form of dispersal movements. Such movements are often assumed to be diffusive (Okubo and Levin 2013), but sometimes incorporate elements of taxis (Kareiva and Odell 1987; Lee et al. 2009; Potts and Petrovskii 2017). The resulting models are often systems of reaction–advection–diffusion (RAD) equations, which are amenable to pattern formation analysis via a number of established mathematical techniques (Murray 2003).

An implicit assumption behind these RAD approaches is that the movement processes (advection and diffusion) take place on the same temporal scale as the birth and death processes (reaction). However, many organisms will undergo significant movement over much shorter timescales. For example, many larger animals (e.g. most birds, mammals, and reptiles) will reproduce only once per year, but may rearrange themselves in space quite considerably in the intervening period between natal events. These rearrangements can give rise to emergent phenomena such as the ‘landscape of fear’ (Laundré et al. 2010), aggregations of coexistent species (Murrell and Law 2003), territoriality (Potts and Lewis 2014), home ranges (Briscoe et al. 2002; Börger et al. 2008), and spatial segregation of interacting species (Shigesada et al. 1979).

Indeed, the study of organism movements has led, in the past decade or two, to the emergence of a whole subfield of ecology, dubbed ‘movement ecology’ (Nathan et al. 2008; Nathan and Giuggioli 2013). This is gaining increasing attention by both statisticians (Hooten et al. 2017) and empirical ecologists (Kays et al. 2015; Hays et al. 2016), in part driven by recent rapid technological advances in biologging (Williams et al., in review). Often, a stated reason for studying movement is to gain insight into space-use patterns (Vanak et al. 2013; Avgar et al. 2015; Fleming et al. 2015; Avgar et al. 2016). Yet despite this, we lack a good understanding of the spatial pattern formation properties of animal movement models over timescales where birth and death effects are minimal.

To help rectify this situation, we introduce here a class of models that focuses on one particular type of movement: taxis of a population in response to the current or recent presence of foreign populations. This covers several ideas within the ecological literature. One is the movement of a species away from areas where predator or competitor species reside, often dubbed the ‘landscape of fear’ (Laundré et al. 2010; Gallagher et al. 2017). The opposing phenomenon is that of predators moving towards prey, encapsulated in prey-taxis models (Kareiva and Odell 1987; Lee et al. 2009). Many species exhibit mutual avoidance, which can be either inter-species avoidance or intra-species avoidance. The latter gives rise to territoriality, and there is an established history of modelling efforts devoted to its study (Adams 2001; Lewis and Moorcroft 2006; Potts and Lewis 2014). Likewise, some species exhibit mutual attraction due to benefits of coexistence (Murrell and Law 2003; Kneitel and Chase 2004; Vanak et al. 2013). Since some of these phenomena are inter-specific and others are intra-specific, we use the word ‘population’ to mean a group of organisms that are all modelled using the same equation, noting once and for all that ‘population’ may be used to mean an entire species (for modelling inter-species interactions, e.g. the landscape of fear), or it may refer to a group within a single species (for intra-species interactions, e.g. territoriality).

There are various processes by which one population can sense the presence of others. One is by directly sensing organism presence by sight or touch. However, it is perhaps more common for the presence of others to be advertised indirectly. This could either be due to marks left in the landscape, a process sometimes known as stigmergy (Giuggioli et al. 2013), or due to memory of past interactions (Fagan et al. 2013; Potts and Lewis 2016a). We show here that these three interaction processes (direct, stigmergic, memory) can all be subsumed under a single modelling framework.

The resulting model is a system of *N* diffusion–taxis equations, one for each of *N* populations. We analyse this system using a combination of linear pattern formation analysis (Turing 1952), energy functionals (nonlinear), and numerical bifurcation analysis. We classify completely the pattern formation properties for $N=2$, noting that here only stationary patterns can form. For $N=3$, we show that, as well as there being parameter regimes where stationary patterns emerge, oscillatory patterns can emerge for certain parameter values, where patterns remain transient and never settle to a steady state. In these regimes, we observe both periodic behaviour and behaviour where the period is much less regular. These irregular regimes emerge through a sequence of period-doubling bifurcations, a phenomenon often associated with the emergence of chaos.

The fact that inter-population taxis processes can give rise to perpetually changing, possibly chaotic, spatial patterns is a key insight into the study of species distributions. Researchers often look to explain such transient spatial patterns by examining changes in the underlying environment. However, we show that continually changing patterns can emerge without the need to impose any environmental effect. As such, our study highlights the importance of understanding inter-population movement responses for gaining a full understanding of the spatial distribution of ecological communities, and helps link movement ecology to population dynamics in a non-speculative way.

## The Modelling Framework

Our general modelling framework considers *N* populations, each of which has a fixed overall size. For each population, the constituent individuals move in space through a combination of a diffusive process and a tendency to move towards more attractive areas and away from those that are less attractive. Denoting by $u_i(\mathbf{x},t)$ the probability density function of population *i* at time *t* ($i \in \{1,\dots ,N\}$), and by $A_i(\mathbf{x},t)$ the attractiveness of location $\mathbf{x}$ to members of population *i* at time *t*, we construct the following movement model

1 $$\begin{aligned} \frac{\partial u_i}{\partial t} = D_i\nabla ^2 u_i - c_{i} \nabla \cdot \left( u_i \nabla A_i \right) , \end{aligned}$$

where $D_i>0$ is the magnitude of the diffusive movement of population *i* and $c_i\ge 0$ is the magnitude of the drift tendency towards more attractive parts of the landscape.

Here, we assume that the attractiveness of a point $\mathbf{x}$ on the landscape at time *t* is determined by the presence of individuals from other populations. We look at three scenarios. For some organisms, particularly very small ones such as amoeba, there may be sufficiently many individuals constituting each population so that the probability density function is an accurate descriptor of the number of individuals present at each part of space. This is Scenario 1. In this case, the attractiveness of a part of space to population *i* may simply be proportional to the weighted sum of the probability density functions of all the other populations, or possibly a locally averaged probability density. In other words

2 $$\begin{aligned} \mathbf{Scenario}\,\mathbf{1{:}}\,A_i(\mathbf{x},t) = \sum _{j\ne i} a_{ij} {\bar{u}}_j(\mathbf{x},t), \end{aligned}$$

where $a_{ij}$ are constants, which can be either positive, if population *i* benefits from the presence of population *j*, or negative, if population *i* seeks to avoid population *j*, and

3 $$\begin{aligned} {\bar{u}}_j(\mathbf{x},t) = \frac{1}{|C_\mathbf{x}|}\int _{C_\mathbf{x}}u_j(\mathbf{z},t)\mathrm{d}{} \mathbf{z}, \end{aligned}$$

where $C_\mathbf{x}$ is a small neighbourhood of $\mathbf{x}$ and $|C_\mathbf{x}|$ is the Lebesgue measure of $C_\mathbf{x}$. The importance of this spatial averaging will become apparent in Sect. 3.

For larger organisms (e.g. mammals, birds, reptiles, etc.), individuals may be more spread out on the landscape. Here, the presence may be advertised by one of two processes (Scenarios 2 and 3). In Scenario 2, we model individuals as leaving marks on the landscape (e.g. urine, faeces, footprints etc.) to which individuals of the other populations respond. Denoting by $p_i$ the presence of marks that are foreign to population *i*, we can model this using the following differential equation (cf. Lewis and Murray 1993; Lewis and Moorcroft 2006; Potts and Lewis 2016b)

4 $$\begin{aligned} \frac{\partial p_i}{\partial t} = \sum _{j\ne i}\alpha _{ij} u_j-\mu p_i, \end{aligned}$$

where $\mu >0$ and $\alpha _{ij} \in {{\mathbb {R}}}$ are constants. If $\alpha _{ij}>0$ (resp. $\alpha _{ij}<0$), then population *i* is attracted towards (resp. repelled away from), population *j*. In this scenario, we model $A_i(\mathbf{x},t)$ as a spatial averaging of $p_i(\mathbf{x},t)$ so that

5 $$\begin{aligned} \mathbf{Scenario}\,\mathbf{2{:}}\,A_i(\mathbf{x},t) = {\bar{p}}_i(\mathbf{x},t), \end{aligned}$$

where ${\bar{p}}_i(\mathbf{x},t)$ is defined in an analogous way to ${\bar{u}}_j(\mathbf{x},t)$ in Eq. (3).

Finally, Scenario 3 involves individuals remembering places where they have had recent encounters with individuals of another population, and moving in a manner consistent with a cognitive map. We assume here that individuals within a population are able to transmit information between themselves so that they all share common information regarding the expected presence of other populations, which we denote by $k_i(\mathbf{x},t)$ for population *i*. This can be modelled as follows (cf. Potts and Lewis 2016a)

6 $$\begin{aligned} \frac{\partial k_i}{\partial t} = \sum _{j\ne i}\beta _{ij} u_iu_j-(\zeta +\nu u_i) k_i, \end{aligned}$$

where $\nu > 0, \zeta \ge 0$, and $\beta _{ij}\in {{\mathbb {R}}}$ are constants. Here, $\beta _{ij}$ refers to the tendency for animals from population *i* to remember a spatial location, given an interaction with an individual from population *j*, $\zeta$ is the rate of memory decay, and $\nu$ refers to the tendency for animals from population *i* to consider a location not part of *j*’s range if individuals from *i* visit that location without observing an individual from *j* there. See Potts and Lewis (2016a) more explanation of the motivation and justification for the functional form in Eq. (6), in the context of avoidance mechanisms.

In this scenario, we model $A_i(\mathbf{x},t)$ as a spatial averaging of $k_i(\mathbf{x},t)$ so that

7 $$\begin{aligned} \mathbf{Scenario}\,\mathbf{3{:}}\,A_i(\mathbf{x},t) = {\bar{k}}_i(\mathbf{x},t), \end{aligned}$$

where ${\bar{k}}_i(\mathbf{x},t)$ is defined in an analogous way to ${\bar{u}}_j(\mathbf{x},t)$ in Eq. (3).

Note the similarity between Scenarios 2 and 3 and the idea of a “landscape of fear”, which has become increasingly popular in the empirical literature (Laundré et al. 2010). The landscape of fear invokes the idea that there are certain parts of space that individuals in a population tend to avoid because they perceive those areas to have a higher risk of aggressive interactions (either due to predation or competition). The degree to which this danger is perceived across space creates a spatial distribution of fear, and animals may be modelled as advecting down the gradient of this distribution.

## General Results in 1D

Although our modelling framework can be defined in arbitrary dimensions, we will focus our analysis on the following 1D version of Eq. (1)

8 $$\begin{aligned} \frac{\partial u_i}{\partial t} = D_i\frac{\partial ^2 u_i}{\partial x^2} - c_{i} \frac{\partial }{\partial x}\left( u_i \frac{\partial A_i}{\partial x} \right) . \end{aligned}$$

We also work on a line segment, so that $x \in [0,L]$ for some $L>0$.

It is convenient for analysis to assume that, for Scenarios 2 and 3, the quantities $p_i({x},t)$ and $k_i({x},t)$ equilibrate much faster than $u_i({x},t)$, so we can make the approximations ${\partial p_i}/{\partial t}\approx 0$ and ${\partial k_i}/{\partial t}\approx 0$. Making the further assumption that there is no memory decay ($\zeta =0$ in Eq. 6), which turns out later to be convenient for unifying the three scenarios, we have the following approximate versions of Eqs. (5) and (7)

9 $$\begin{aligned} \mathbf{Scenario}\,\mathbf{2{:}}\,A_i({x},t)&\approx \sum _{j\ne i}\frac{\alpha _{ij}}{\mu } {\bar{u}}_j({x},t), \end{aligned}$$

10 $$\begin{aligned} \mathbf{Scenario}\,\mathbf{3{:}}\,A_i({x},t)&\approx \sum _{j\ne i}\frac{\beta _{ij}}{\nu } {\bar{u}}_j({x},t). \end{aligned}$$

We non-dimensionalise our system by setting ${\tilde{u}}_i=Lu_i$, ${\tilde{x}}=x/L$, ${\tilde{t}}=tD_1/L^2$, $d_i=D_i/D_1$ and

11 $$\begin{aligned} \gamma _{ij}= {\left\{ \begin{array}{ll} \frac{c_ia_{ij}}{LD_1},&{}\quad \text{ in } \text{ Scenario } \text{1 }, \\ \frac{c_i\alpha _{ij}}{\mu LD_1},&{}\quad \text{ in } \text{ Scenario } \text{2 }, \\ \frac{c_i\beta _{ij}}{\nu LD_1},&{}\quad \text{ in } \text{ Scenario } \text{3 }. \end{array}\right. } \end{aligned}$$

Then, dropping the tildes over ${\tilde{u}}_i$, ${\tilde{x}}$, and ${\tilde{t}}$ for notational convenience, we obtain the following non-dimensional model for space use

12 $$\begin{aligned} \frac{\partial u_i}{\partial t}&= d_i\frac{\partial ^2 u_i}{\partial x^2} - \frac{\partial }{\partial x}\left( u_i \sum _{j\ne i}\gamma _{ij}\frac{\partial {\bar{u}}_j}{\partial x} \right) , \end{aligned}$$

where $d_1=1$, by definition.

For simplicity, we assume that boundary conditions are periodic, so that

13 $$\begin{aligned} u_i(0,t)=u_i(1,t). \end{aligned}$$

With this identification in place, we can define the 1D spatial averaging kernel from Eq. (3) to be $C_x=\{z \in [0,1] | (x-\delta ) (\text{ mod } 1)<z<(x+\delta ) (\text{ mod } 1)\}$ for $0<\delta \ll 1$. Here, $z(\text{ mod } 1)$ is used so as to account for the periodic boundary conditions and is defined to be the unique real number $z' \in [0,1)$ such that $z-z' \in {\mathbb {Z}}$. Then, Eq. (3) becomes

14 $$\begin{aligned} {\bar{u}}_j = \frac{1}{2\delta }\int _{(x-\delta )(\text{ mod } 1)}^{(x+\delta )(\text{ mod } 1)} u_j(z,t)\mathrm{d}z. \end{aligned}$$

Finally, since $u_i(x,t)$ are probability density functions of *x*, defined on the interval $x \in [0,1]$, we also have the integral condition

15 $$\begin{aligned} \int _0^1 u_i(x,t)\mathrm{d}x=1. \end{aligned}$$

This condition means that we have a unique spatially homogeneous steady state, given by $u_i^*(x)=1$ for all $i \in \{1,\dots ,N\}, x \in [0,1]$. Our first task for analysis is to see whether this steady state is unstable to non-constant perturbations.

We set $\mathbf{w}(x,t)=(u_1-1,\dots ,u_N-1)^\mathrm{T}=(u_{1}^{(0)},\dots ,u_{N}^{(0)})^\mathrm{T}\exp (\sigma t + \mathrm{i}\kappa x)$, where $u_1^{(0)},...,u_N^{(0)}$ and $\sigma ,\kappa$ are constants, and the superscript T denotes matrix transpose. By neglecting nonlinear terms, Eq. (12) becomes

16 $$\begin{aligned} \sigma \mathbf{w}=\kappa ^2 M(\kappa ,\delta )\mathbf{w}, \end{aligned}$$

where $M(\kappa ,\delta )=[M_{ij}(\kappa ,\delta )]_{i,j}$ is a matrix with

17 $$\begin{aligned} M_{ij}(\kappa ,\delta )={\left\{ \begin{array}{ll} -d_i, &{}\quad \text{ if } i=j, \\ \gamma _{ij}\text{ sinc }(\kappa \delta ), &{}\quad \text{ otherwise, } \end{array}\right. } \end{aligned}$$

where $\text{ sinc }(\xi )=\sin (\xi )/\xi$. Therefore, patterns form whenever there is some $\kappa$ such that there is an eigenvalue of $M(\kappa ,\delta )$ with positive real part.

It is instructive to examine the limit case $\delta \rightarrow 0$. Here

18 $$\begin{aligned} M_{ij}(\kappa ,0)={\left\{ \begin{array}{ll} -d_i, &{}\quad \hbox { if}\ i=j \\ \gamma _{ij}, &{}\quad \text{ otherwise. } \end{array}\right. } \end{aligned}$$

so $M_{ij}(\kappa ,0)$ is, in fact, independent of $\kappa$, and so we define the constant matrix $M_0=[M_{ij}(\kappa ,0)]_{i,j}$. When $\delta \rightarrow 0$, there are two cases pertinent to pattern formation:

1. All the eigenvalues of $M_0$ have negative real part, in which case no patterns form.
2. At least one eigenvalue of $M_0$ has positive real part, in which case the dominant eigenvalue of $\kappa ^2 M_0$ is an increasing function of $\kappa$. Therefore, patterns can form at arbitrarily high wavenumbers. In other words, the pattern formation problem is ill-posed.

**Fig. 1:**
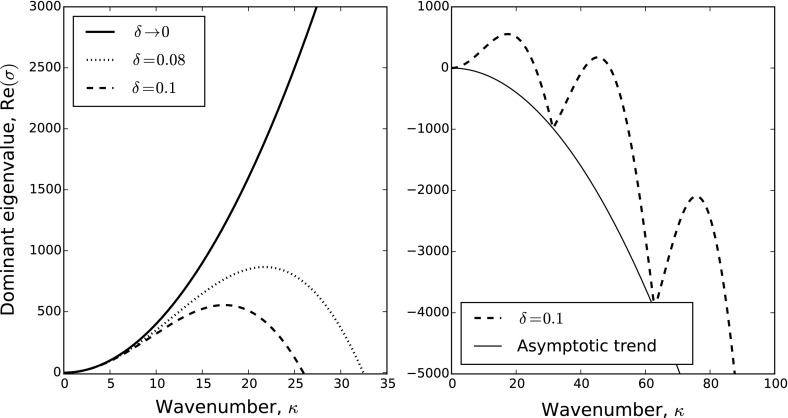
Example dispersion relations. Here we give dispersion relations for the system described by Eq. (12) with $N=2$, $d_i=1$, and $\gamma _{ij}=-5$ for all *i*, *j*. In the left-hand panel, we examine three values of $\delta$, showing that, for $\delta \rightarrow 0$, the dispersion relation is monotonic, but this monotonicity is tamed by setting $\delta >0$. In the right-hand panel, we extend the dispersion relation plot for $\delta =0.1$ to a larger range of $\kappa$ values, together with the analytically derived asymptotic trend

The problem posed by point (2) above can often be circumvented by using a strictly positive $\delta$. For example, Fig. 1 shows the dispersion relation (plotting the dominant eigenvalue against $\kappa$) for a simple case where $N=2$, $d_i=1$, $\gamma _{ij}=-5$ for all *i*, *j*, and $\delta$ is varied. In this example, the dominant eigenvalue is real for all $\kappa$. We see that, for $\delta \rightarrow 0$, the dispersion relation is monotonically increasing. However, a strictly positive $\delta$ means the eigenvalues are $\kappa ^2[-2 \pm 5\text{ sinc }(\kappa \delta )]/2$, which is asymptotically $\sigma \approx -\kappa ^2$ as $\kappa \rightarrow \infty$. Hence, the dominant eigenvalue is positive only for a finite range of $\kappa$ values, as long as $\delta >0$.

The fact that the pattern formation problem is ill-posed for $\delta \rightarrow 0$ suggests that classical solutions may not exist in this case. This phenomenon is not new and has been observed in very similar systems studied by Briscoe et al. (2002), Potts and Lewis (2016a, b). More generally, there are various studies that deal with regularisation of such ill-posed problems in slightly different contexts using other techniques, which incorporate existence proofs (e.g. Padrón 1998, 2004). We therefore conjecture that classical solutions do exist for the system given by Eq. (12) in the case where $\delta >0$, and the numerics detailed in this paper give evidence to support this. However, we do not prove this conjecture here, since it is a highly non-trivial question in general, and the purpose of this paper is just to introduce the model structure and investigate possible types of patterns that could arise. Nonetheless, it is an important subject for future research. In the next two sections, we will examine specific cases where $N=2$ and $N=3$.

## The Case of Two Interacting Populations ($N=2$)

When $N=2$, the system given by Eqs. (12, 14, 15) is simple enough to categorise its linear pattern formation properties in full. Here

19 $$\begin{aligned} M(\kappa ,\delta )=\left( \begin{array}{cc} -1 &{}\quad \gamma _{12}\text{ sinc }(\kappa \delta ) \\ \gamma _{21}\text{ sinc }(\kappa \delta ) &{}\quad -d_2 \end{array} \right) . \end{aligned}$$

The eigenvalues of $M(\kappa ,\delta )$ are therefore

20 $$\begin{aligned} \sigma (\kappa ) = \frac{-(1+d_2)\pm \sqrt{(1+d_2)^2+4[\gamma _{12}\gamma _{21}\text{ sinc }^2(\kappa \delta )-d_2]}}{2}. \end{aligned}$$

Notice first that if $\sigma (\kappa )$ is not real, then the real part is $\text{ Re }[\sigma (\kappa )]=-(1+d_2)/2$, which is always negative, since $d_2>0$. Hence, patterns can only form when $\sigma (\kappa )\in {{\mathbb {R}}}$, meaning that the discriminant, $\varDelta =(1+d_2)^2+4[\gamma _{12}\gamma _{21}\text{ sinc }^2(\kappa \delta )-d_2]$, must be positive. In addition, $\sigma (\kappa )>0$ only when $\varDelta >(1+d_2)^2$. This occurs whenever $\gamma _{12}\gamma _{21}\text{ sinc }^2(\kappa \delta ) > d_2$. Since the maximum value of $\text{ sinc }^2(\kappa \delta )$ is 1, which is achieved at $\kappa =0$, we arrive at the following necessary criterion for pattern formation, which is also sufficient if we either drop the boundary conditions or take the $\delta \rightarrow 0$ limit

21 $$\begin{aligned} \gamma _{12}\gamma _{21} > d_2. \end{aligned}$$

Furthermore, any patterns that do form are stationary patterns, since the eigenvalues are always real if their real part is positive.

There are three distinct biologically relevant situations, which correspond to different values of $\gamma _{12}$ and $\gamma _{21}$, as follows

1. Mutual avoidance: $$\gamma _{12},\gamma _{21}<0$$
2. Mutual attraction: $$\gamma _{12},\gamma _{21}>0$$
3. Pursue-and-avoid: $\gamma _{12}<0<\gamma _{21}$ or $\gamma _{21}<0<\gamma _{12}$

**Fig. 2:**
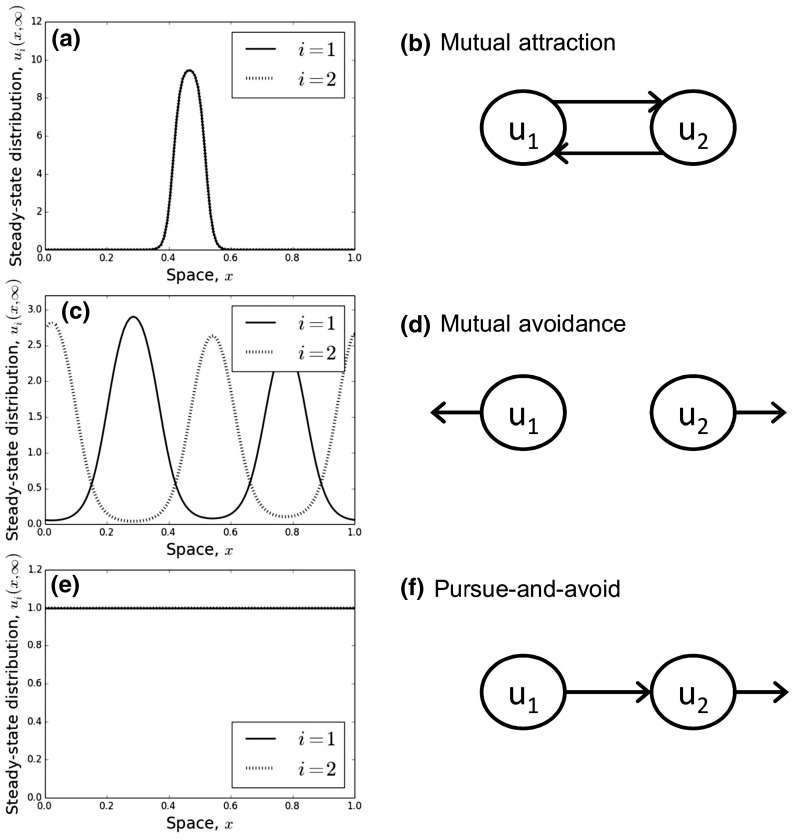
Dynamics for a two-species system. Here, there are three cases: mutual attraction, mutual avoidance, and pursue-and-avoid. **a** The steady state of a model of mutual attraction, with $\gamma _{12}=\gamma _{21}=2$, and $\delta =0.1$, with a schematic of this situation in **b**. **c** The steady state of a mutual avoidance model with $\gamma _{12}=\gamma _{21}=-2$, and $\delta =0.1$, with corresponding schematic in **d**. **e** The steady state of a pursue-and-avoid model (where patterns never form) with $\gamma _{12}=2, \gamma _{21}=-2$ and $\delta =0.1$, with corresponding schematic in **f**

There are also the edge cases where $\gamma _{12}=0$ or $\gamma _{21}=0$, which we will not focus on. Notice that the ‘pursue-and-avoid’ case cannot lead to the emergence of patterns (Fig. 2e), as it is inconsistent with the inequality in (21). However, the other two situations can.

Mutual avoidance leads to spatial segregation if Inequality (21) is satisfied (Fig. 2c). Some previous models of territory formation in animal populations by the present authors have a very similar form to the mutual avoidance model here, so we refer to Potts and Lewis (2016a, b) for details of this situation. Mutual attraction leads to aggregation of both populations in a particular part of space, whose width roughly corresponds to the width of the spatial averaging kernel, $(x-\delta ,x+\delta )$ (Fig. 2a), as long as Inequality (21) is satisfied.

The characterisation of between-population movement responses into ‘mutual avoidance’, ‘mutual attraction’, and ‘pursue-and-avoid’ enables us to categorise examples of the system in Eqs. (12, 14, 15) by means of a simple schematic diagram. We construct one node for each population, ensuring that no three distinct nodes are in a straight line. Then, an arrow is added from node *i* to node *j* if $\gamma _{ij}>0$. If $\gamma _{ij}<0$, an arrow is added from node *i* in the direction anti-parallel to the line from node *i* to node *j*. These diagrams allow us to see quickly the qualitative relationship between the populations (see Fig. 2b,d,f for the $N=2$ case and Fig. 4b for some examples in the $N=3$ case).

### An Energy Functional Approach to Analysing Patterns

We can gain qualitative understanding of the patterns observed in Fig. 2a–d via use of an energy functional approach, by assuming $\gamma _{1,2}=\gamma _{2,1}=\gamma$, and $d_2=1$. In particular, this approach gives a mathematical explanation for the appearance of aggregation patterns when $\gamma >0$ and segregation patterns when $\gamma <0$. The results rely on the assumption that, for all *i*, $u_i(x,0)> 0$ implies $u_i(x,t)> 0$ for all *t*, which can be shown by the application of a comparison theorem to Eqs. (8, 13), assuming ${\partial }A_i(x)/{\partial }x$ is bounded. Throughout this section, our spatial coordinates will be defined on the quotient space $[0,1]/\{0,1\}$, which is consistent with our use of periodic boundary conditions.

Our method makes use of the following formulation of Eq. (12)

22 $$\begin{aligned} \frac{\partial u_i}{\partial t}&= \frac{\partial }{\partial x}\left[ u_i\frac{\partial }{\partial x}\left( d_i\ln (u_i) -\sum _{j\ne i}\gamma _{ij}{{\mathcal {K}}}*u_j \right) \right] , \end{aligned}$$

and also the energy functional

23 $$\begin{aligned} E(u_1,u_2)=\int _0^1\{u_1[2\ln (u_1)-\gamma {\mathcal K} *u_2]+u_2[2\ln (u_2)-\gamma {{\mathcal {K}}} *u_1]\}\mathrm{d}x, \end{aligned}$$

where ${{\mathcal {K}}}(x)$ is a bounded function (i.e. $\left||{{\mathcal {K}}}\right||_{\infty } < \infty$), symmetric about $x=0$ on the domain $[0,1]/\{0,1\}$, with $\left||{\mathcal K}\right||_1=1$, and $*$ denotes the following spatial convolution

24 $$\begin{aligned} {{\mathcal {K}}} *u_i(x,t) = \int _0^1 {\mathcal K}(x-y) u_i(y,t)\mathrm{d}y. \end{aligned}$$

In our situation, Eq. (14) implies that ${\mathcal K}(x)=1/(2\delta )$ for $-\delta< x < \delta (\text{ mod } 1)$ and ${{\mathcal {K}}}(x)=0$ for $\delta \le x \le 1-\delta$. We consider solutions $u_1(x,t)$ and $u_2(x,t)$ that are continuous functions of *x* and *t*.

We show that the energy functional from Eq. (23) decreases over time to a minimum, which represents the steady-state solution of the system. The monotonic decrease of *E* over time is shown as follows

25 $$\begin{aligned} \frac{\partial E}{\partial t}&= \int _0^1\left\{ \frac{\partial u_1}{\partial t}[2\ln (u_1)-\gamma {{\mathcal {K}}} *u_2]+\frac{\partial u_2}{\partial t}[2\ln (u_2)-\gamma {{\mathcal {K}}} *u_1]\right\} \mathrm{d}x \nonumber \\&\quad +\int _0^1 \left[ 2 \frac{\partial u_1}{\partial t}+2 \frac{\partial u_2}{\partial t}-\gamma u_1 {{\mathcal {K}}} *\frac{\partial u_2}{\partial t}-\gamma u_2 {{\mathcal {K}}} *\frac{\partial u_1}{\partial t}\right] \mathrm{d}x\nonumber \\&=\int _0^1\left\{ 2 \frac{\partial u_1}{\partial t}+2 \frac{\partial u_2}{\partial t}+\frac{\partial u_1}{\partial t}[2\ln (u_1)-2\gamma {{\mathcal {K}}}*u_2]+\frac{\partial u_2}{\partial t}[2\ln (u_2)-2\gamma {{\mathcal {K}}}*u_1]\right\} \mathrm{d}x \nonumber \\&=2\int _0^1\biggl \{\frac{\partial }{\partial x}\left[ u_1\frac{\partial }{\partial x}\left( \ln (u_1) -\gamma {{\mathcal {K}}}*u_2\right) \right] [1+\ln (u_1)-\gamma {{\mathcal {K}}}*u_2]\nonumber \\&\quad +\frac{\partial }{\partial x}\left[ u_2\frac{\partial }{\partial x}\left( \ln (u_2) -\gamma {{\mathcal {K}}}*u_1\right) \right] [1+\ln (u_2)-\gamma {{\mathcal {K}}}*u_1]\biggr \}\mathrm{d}x \nonumber \\&=2\biggl [u_1\frac{\partial }{\partial x}(\ln (u_1)-\gamma {{\mathcal {K}}}*u_2)(1+\ln (u_1)-\gamma {{\mathcal {K}}}*u_2)\nonumber \\&\quad +u_2\frac{\partial }{\partial x}(\ln (u_2)-\gamma {{\mathcal {K}}}*u_1)(1+\ln (u_2)-\gamma {{\mathcal {K}}}*u_1)\biggr ]^1_0\nonumber \\&\quad -2 \int _0^1\biggl \{\left[ u_1\frac{\partial }{\partial x}(\ln (u_1)-\gamma {{\mathcal {K}}}*u_2)\right] \frac{\partial }{\partial x}(\ln (u_1)-\gamma {{\mathcal {K}}}*u_2)\nonumber \\&\quad +\left[ u_2\frac{\partial }{\partial x}(\ln (u_2)-\gamma {{\mathcal {K}}}*u_1)\right] \frac{\partial }{\partial x}(\ln (u_2)-\gamma {{\mathcal {K}}}*u_1)\biggr \}\mathrm{d}x \nonumber \\&=-2 \int _0^1\biggl \{\left[ u_1\frac{\partial }{\partial x}(\ln (u_1)-\gamma {{\mathcal {K}}}*u_2)\right] \frac{\partial }{\partial x}(\ln (u_1)-\gamma {{\mathcal {K}}}*u_2)\nonumber \\&\quad +\left[ u_2\frac{\partial }{\partial x}(\ln (u_2)-\gamma {{\mathcal {K}}}*u_1)\right] \frac{\partial }{\partial x}(\ln (u_2)-\gamma {{\mathcal {K}}}*u_1)\biggr \}\mathrm{d}x \nonumber \\&=-2 \int _0^1\left\{ u_1\left[ \frac{\partial }{\partial x}(\ln (u_1)-\gamma {{\mathcal {K}}}*u_2)\right] ^2+u_2\left[ \frac{\partial }{\partial x}(\ln (u_2)-\gamma {{\mathcal {K}}}*u_1)\right] ^2\right\} \mathrm{d}x \nonumber \\&\le 0. \end{aligned}$$

Here, the first equality uses Eq. (23), the second uses the fact that $\int _0^1 f(x){{\mathcal {K}}}*h(x)\mathrm{d}x=\int _0^1 h(x){{\mathcal {K}}}*f(x)\mathrm{d}x$ as long as ${{\mathcal {K}}}(x)$ is symmetric about 0 in $[0,1]/\{0,1\}$, and also requires that $\gamma _{1,2}=\gamma _{2,1}=\gamma$, the third uses Eq. (22), the fourth is integration by parts, the fifth uses the fact that $u_i(0)=u_i(1)$ and ${{\mathcal {K}}}*u_i(0)={{\mathcal {K}}}*u_i(1)$ for $i \in \{1,2\}$ (i.e. periodic boundary conditions, Eq. 13), the sixth is just a rearrangement, and the inequality at the end uses the fact that $u_i(x,t)> 0$ for all *i*, *x*, *t*. In all, Eq. (25) shows that $E(u_1,u_2)$ is decreasing over time. The following shows that $E(u_1,u_2)$ is bounded below

26 $$\begin{aligned} E(u_1,u_2)&=2\int _0^1 [u_1\ln (u_1)+u_2\ln (u_2)]\mathrm{d}x-\int _0^1 [u_1 {{\mathcal {K}}}*u_1+u_2 {{\mathcal {K}}}*u_2]\mathrm{d}x\nonumber \\&\ge -4\mathrm{e}^{-1}-\int _0^1 [u_1 {{\mathcal {K}}}*u_1+u_2 {{\mathcal {K}}}*u_2]\mathrm{d}x\nonumber \\&\ge -4\mathrm{e}^{-1}-\left||u_1\right||_1\left||{{\mathcal {K}}}*u_1\right||_\infty -\left||u_2\right||_1\left||{{\mathcal {K}}}*u_2\right||_\infty \nonumber \\&\ge -4\mathrm{e}^{-1}-\left||u_1\right||_1\left||{{\mathcal {K}}}\right||_\infty \left||u_1\right||_1-\left||u_2\right||_1\left||{{\mathcal {K}}}\right||_\infty \left||u_2\right||_1\nonumber \\&\ge -4\mathrm{e}^{-1}-2\left||{{\mathcal {K}}}\right||_\infty . \end{aligned}$$

Here, the first inequality uses the fact that $\text{ inf }_{u_i\ge 0} \{u_i \ln (u_i)\}=-\mathrm{e}^{-1}$, the second uses Hölder’s inequality, the third uses Young’s inequality, and the fourth uses the fact that $\left||u_1\right||_1=1$ (Eq. 15). For the absence of doubt, the definition $\left||f\right||_p=\left( \int _0^1|f(x,t)|^p\mathrm{d}x\right) ^{1/p}$, for $p \in [1,\infty ]$, is used throughout (26). Again, note that the inequality $u(x,t)>0$ is required for the sequence of inequalities in (26) to hold.

The inequalities in (25) and (26) together demonstrate that $E(u_1,u_2)$ moves towards a minimum as $t\rightarrow \infty$, which is given at the point where $\frac{\partial E}{\partial t}=0$. The latter equation is satisfied when the following two conditions hold

27 $$\begin{aligned} \ln (u_1)-\gamma {{\mathcal {K}}}*u_2&=\eta _1, \end{aligned}$$

28 $$\begin{aligned} \ln (u_2)-\gamma {{\mathcal {K}}}*u_1&=\eta _2, \end{aligned}$$

where $\eta _1$ and $\eta _2$ are constants.

**Fig. 3:**
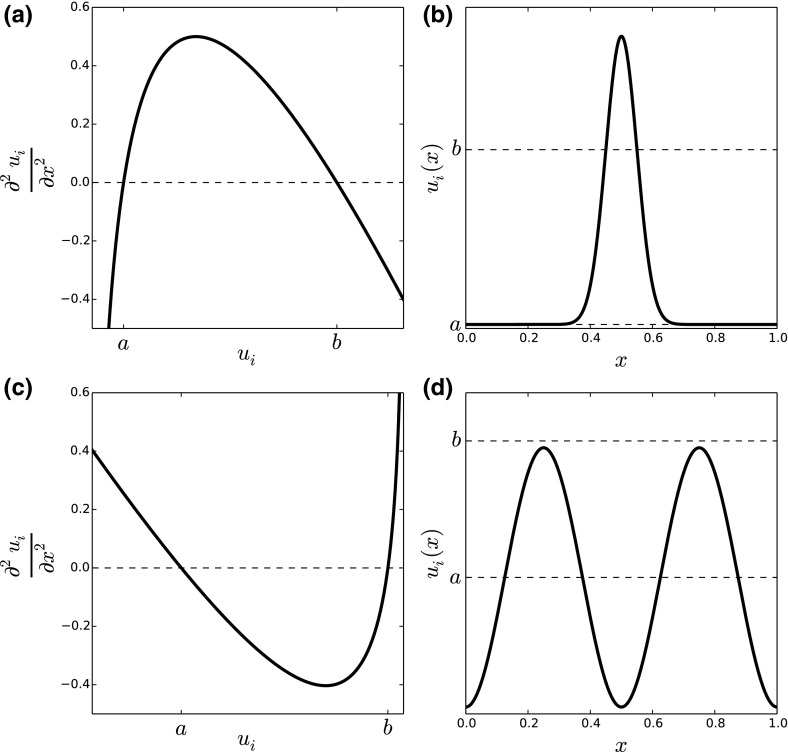
Understanding the patterns from Fig. 2 using energy functionals. **a** An example of $\frac{\partial ^2 u_i}{\partial x^2}$ as a function of $u_i$ (Eq. 32) when the energy is minimised (Eqs. 27–28) and the moment closure approximation from Eq. (31) is applied, for the aggregation case, $u_1 \approx u_2$. We see that $\frac{\partial ^2 u_i}{\partial x^2}$ is positive for $a<u_i<b$ and negative when $u_i<a$ or $u_i>b$. There are various possible smooth solutions, $u_i(x,\infty )$, that satisfy this property. **b** An example corresponding qualitatively to Fig. 2a. **c**, **d** Are analogous to **a** and **b**, respectively, but for the situation where we have segregation, so $u_1 \approx 2-u_2$. Note that **d** qualitatively resembles Fig. 2c

Equations (27–28) can be used to give qualitative properties of the long-term distribution of the system in Eqs. (12, 14, 15) for $N=2$ and $\gamma _{1,2}=\gamma _{2,1}=\gamma$. First, by differentiating Eqs. (27–28) with respect to *x*, we find that

29 $$\begin{aligned} \frac{\partial u_1}{\partial x}\frac{1}{u_1}=\gamma \frac{\partial }{\partial x}({{\mathcal {K}}}*u_2), \end{aligned}$$

30 $$\begin{aligned} \frac{\partial u_2}{\partial x}\frac{1}{u_2}=\gamma \frac{\partial }{\partial x}({{\mathcal {K}}}*u_1). \end{aligned}$$

Thus, $\gamma >0$ implies that $\frac{\partial u_1}{\partial x}$ has the same sign as $\frac{\partial }{\partial x}({{\mathcal {K}}}*u_2)$ so any patterns that may form will be aggregation patterns (Fig. 2a, b). Furthermore, $\gamma <0$ implies that $\frac{\partial u_1}{\partial x}$ has the opposite sign to $\frac{\partial }{\partial x}({{\mathcal {K}}}*u_2)$ so any patterns that form will be segregation patterns (Fig. 2c, d).

Second, by making the following moment closure approximation

31 $$\begin{aligned} {{\mathcal {K}}}*u_i \approx u_i+\sigma ^2 \frac{\partial ^2 u_i}{\partial x^2}, \end{aligned}$$

where $\sigma ^2$ is the variance of ${{\mathcal {K}}}(x)$, we can gain insight by examining the plot of $\frac{\partial ^2 u_i}{\partial x^2}$ against $u_i$ in particular cases. To give an example in the case of aggregation, if $u_1 \approx u_2$ (as in Fig. 2a), then we have $\gamma >0$ by Eqs. (29–30). Equation (28) implies

32 $$\begin{aligned} \sigma ^2 \frac{\partial ^2 u_1}{\partial x^2} \approx \frac{1}{\gamma }[\ln (u_1)-\eta _2]-u_1. \end{aligned}$$

The right-hand side of Eq. (32) has a unique maximum, which is above the horizontal axis as long as $\eta _2<-1-\ln (\gamma )$ (Fig. 3a). In this case, there are two numbers $a,b \in {{\mathbb {R}}}_{>0}$ such that $\frac{\partial ^2 u_i}{\partial x^2}>0$ when $a<u_i<b$ and $\frac{\partial ^2 u_i}{\partial x^2}<0$ for $u_i<a$ or $u_i>b$. A possible curve that satisfies this property is given in Fig. 3b and qualitatively resembles Fig. 2a.

To give an example in the case of segregation ($\gamma <0$), suppose that $u_1\approx 2-u_2$. Then, by a similar argument to the $u_1 \approx u_2$ case, $\frac{\partial ^2 u_i}{\partial x^2}$ has a unique minimum as long as $\eta _2<-1-\ln (-\gamma )-2\gamma$. In this case, there are two numbers $a,b \in {{\mathbb {R}}}$ such that $\frac{\partial ^2 u_i}{\partial x^2}<0$ when $a<u_i<b$ and $\frac{\partial ^2 u_i}{\partial x^2}>0$ for $u_i<a$ or $u_i>b$. A possible curve that satisfies this property is given in Fig. 3d and qualitatively resembles Fig. 2c.

## The Case of Three Interacting Populations ($N=3$)

Although the $N=2$ case only allows for stationary pattern formation [often called a *Turing instability* after Turing (1952)], for $N>2$ we can observe both stationary and oscillating patterns. The latter arise from what is sometimes known as a *wave instability*, where the dominant eigenvalue of $M(\kappa ,\delta )$ is not real but has positive real part, for some $\kappa$. For $N>2$, the situation becomes too complicated for analytic expressions of the eigenvalues to give any meaningful insight [and indeed, these expressions cannot be found for $N>4$ by a classical result of Galois Theory; see Stewart (2015)], so we begin by examining the eigenvalues for certain example cases in the limit $\delta \rightarrow 0$. This involves finding eigenvalues of the matrix $M_0$ given in Eq. (18).

**Fig. 4:**
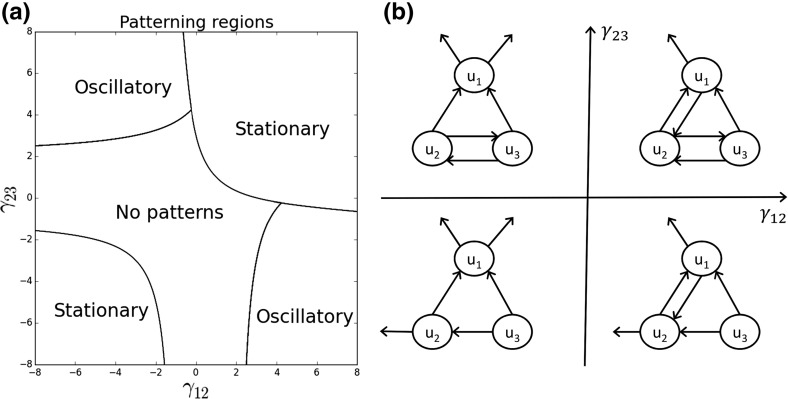
Dynamics for example three-species systems. **a** The pattern formation regions, as predicted by linear analysis, for the system in Eqs. (12, 14, 15) in the case $N=3$, where $d_2=d_3=\gamma _{21}=\gamma _{31}=\gamma _{32}=1$, $\gamma _{13}=-1$, and $\gamma _{12},\gamma _{23}$ are varied. **b** The schematic diagrams of the systems, corresponding to the four quadrants of ($\gamma _{12},\gamma _{23}$)-space

Figure 4 gives an example of how (i) stationary patterns, (ii) oscillatory patterns, and (iii) no patterns can emerge in different regions of parameter space when $N=3$. Here, we have fixed all the $\gamma _{ij}$ except $\gamma _{12}$ and $\gamma _{23}$. Specifically, $d_2=d_3=\gamma _{21}=\gamma _{31}=\gamma _{32}=1$, and $\gamma _{13}=-1$. When $\gamma _{12}<0<\gamma _{23}$, this corresponds to a mutual attraction between populations 2 and 3 with both 2 and 3 pursuing 1 in a pursue-and-avoid situation (Fig. 4b, top-left). When $\gamma _{12},\gamma _{23}>0$, 3 is pursuing 1 in a pursue-and-avoid, whilst 2 is mutually attracted to both 1 and 3 (Fig. 4b, top-right). If $\gamma _{23}<0<\gamma _{12}$, 3 is pursuing both 1 and 2 in a pursue-and-avoid, whilst 1 and 2 are mutually attracting (Fig. 4b, bottom right). Finally, if $\gamma _{12},\gamma _{23}<0$, then 3 is pursuing both 1 and 2 in a pursue-and-avoid and 2 is pursuing 1 in a pursue-and-avoid (Fig. 4b, bottom left).

We solved the system in Eqs. (12–15) for various examples from both the stationary and oscillatory pattern regimes shown in Fig. 4. For this, we used periodic boundary conditions as in Eq. (13). We used a finite difference method, coded in Python, with a spatial granularity of $h=10^{-2}$ and a temporal granularity of $\tau =10^{-5}$. Initial conditions were set to be small random fluctuations from the spatially homogeneous steady state.

**Fig. 5:**
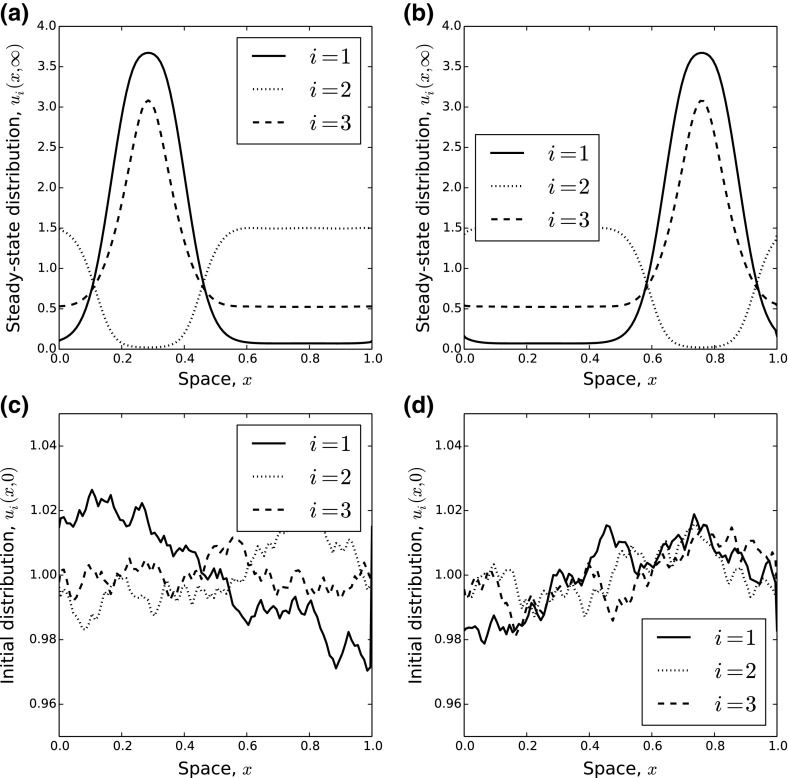
Example three-species systems with stationary distributions. **a**, **b** Two stable steady-state distributions for the system in (12, 14, 15) in the case $N=3$, where $d_2=d_3=\gamma _{21}=\gamma _{31}=\gamma _{32}=1$, $\gamma _{13}=-1$, $\gamma _{12}=\gamma _{23}=-4$, and $\delta =0.1$. **c** [resp. (**d**)] The initial condition that led to the stationary distribution in **a** [resp. (**b**)]

Stationary patterns can give rise to space partitioned into different areas for use by different populations (Fig. 5, Supplementary Video SV1), with differing amounts of overlap. Interestingly, the precise location of the segregated regions depends upon the initial conditions (compare panels (a) and (b) in Fig. 5), but the rough size of the regions appears to be independent of the initial condition (at least for the parameter values we tested). Considering the abundance of individuals as a whole (i.e. $u_1+u_2+u_3$), notice that certain regions of space emerge that contain more animals than others. This is despite the fact that there is no environmental heterogeneity in the model.

The extent to which populations use the same parts of space depends upon the strength of attraction or repulsion. In Fig. 5a, b, the demarcation between populations 1 and 2 is quite stark, owing to the strong avoidance of population 2 by population 1 ($\gamma _{12}=-4$) and a relatively small attraction of population 2 to population 1 ($\gamma _{21}=1$), whereas, although population 1 seeks to avoid 3, the strength of avoidance is smaller ($\gamma _{13}=-1$), but the attraction of population 3 to population 1 is of a similar magnitude ($\gamma _{31}=1$). Therefore, populations 1 and 3 overlap considerably.

**Fig. 6:**
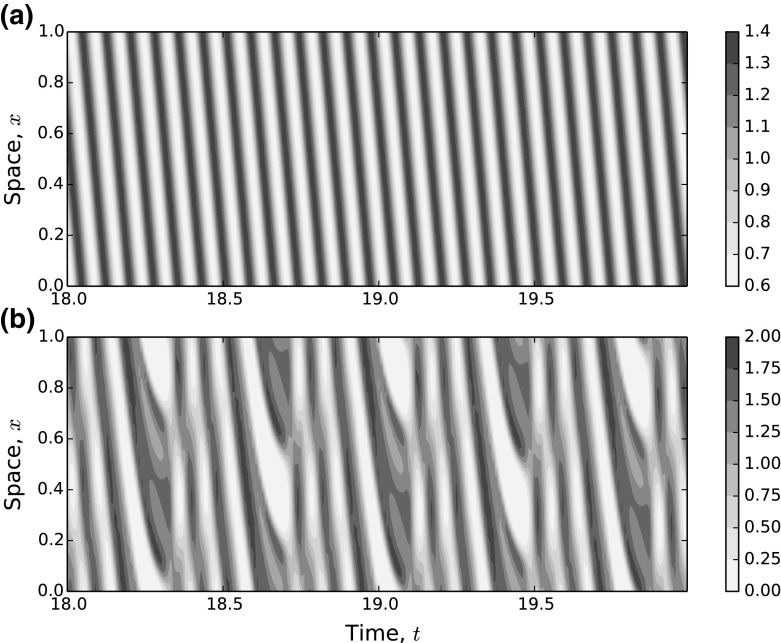
Example three-species systems with oscillatory distributions. Here, we show the change in $u_1(x,t)$ over space and time for two sets of parameter values. Both panels have parameter values identical to the fixed parameters from Fig. 4a, additionally fixing $\gamma _{23}=-4$ and $\delta =0.1$. In **a**, we have $\gamma _{12}=3.3$ and **b** has $\gamma _{12}=4$. We started with random initial conditions and then ran the system to (dimensionless) time $t=20$. The plots display values of $u_1(x,t)$ for $x \in [0,1]$ and $t \in [18,20]$. Plots for $t \in [14,16]$ and $t \in [16,18]$ (not shown) are very similar, indicating that the system has reached its attractor (color figure online)

Oscillatory patterns can be quite complex (Supplementary Video SV2), varying from situations where there appear to be periodic oscillations (Fig. 6a) to those where the periodicity is much less clear (Fig. 6b). To understand their behaviour, we use a method of numerical bifurcation analysis adapted from Painter and Hillen (2011). This method begins with a set of parameters in the region of no pattern formation but close to the region of oscillatory patterns. In particular, we choose parameter values identical to the fixed values for Fig. 4a (i.e. $d_2=d_3=\gamma _{21}=\gamma _{31}=\gamma _{32}=1$, $\gamma _{13}=-1$) and also $\gamma _{23}=-2.5$ and $\gamma _{12}=3$. We then perform the following iterative procedure:

1. Solve the system numerically until $t=10$, by which time the attractor has been reached,
2. Increment $\gamma _{12}$ by a small value (we used 0.005) and set the initial conditions for the next iteration to be the final values of $u_1(x,t)$, $u_2(x,t)$, and $u_3(x,t)$ from the present numerical solution.

This method is intended to approximate a continuous bifurcation analysis. To analyse the resulting patterns, we focus on the value of the system for a fixed point $x=0.5$ and examine how attractor of the system $(u_1(0.5,t),u_2(0.5,t),u_3(0.5,t))$ changes as increase $\gamma _{12}$ into the region of oscillatory patterns.

**Fig. 7:**
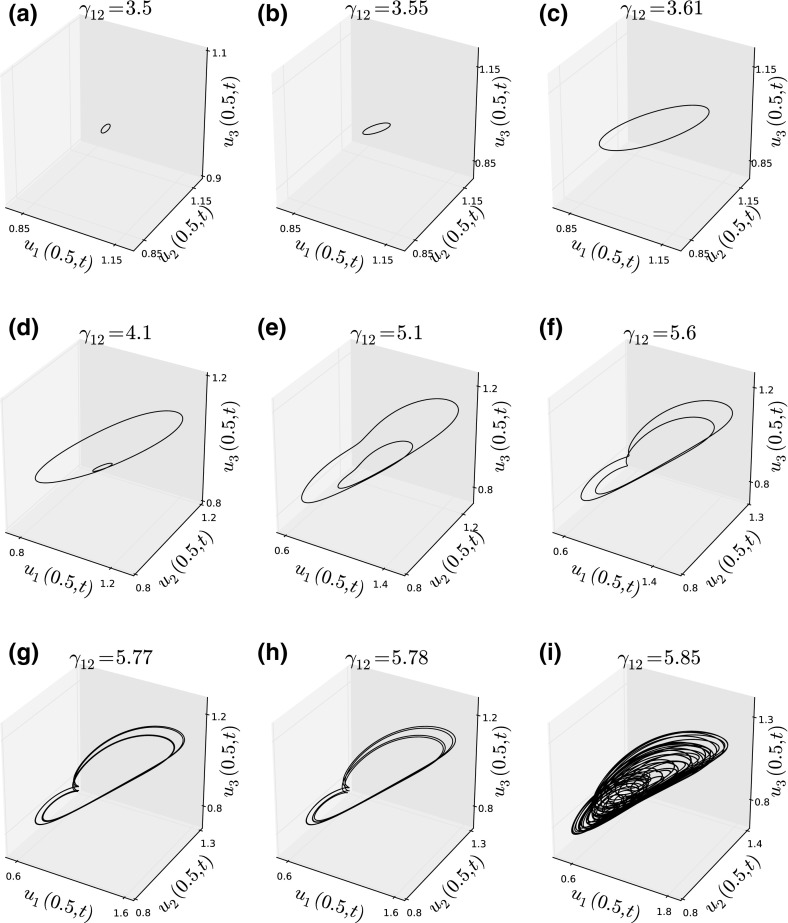
Numerical bifurcation analysis. This sequence of plots shows the attractors just after the system passes through a bifurcation point from a region of no patterns to one of oscillatory patterns. Each panel shows the locus of the point $(u_1(0.5,t),u_2(0.5,t),u_3(0.5,t))$ as time changes for a particular set of parameter values. In all panels, $d_2=d_3=\gamma _{21}=\gamma _{31}=\gamma _{32}=1$, $\gamma _{13}=-1$, and $\gamma _{23}=-2.5$. The value of $\gamma _{12}$ increases from **a** to **i** and is given in the panel title. As $\gamma _{12}$ increases, we observe a sequence of period-doubling bifurcations leading to irregular patterns, suggestive of a chaotic system

Figure 7 shows these attractors for various $\gamma _{12}$ values. First, we observe a small loop appearing just after the system goes through the bifurcation point (Fig. 7a). This loop then grows (Fig. 7b, c) and, when $\gamma _{12}\approx 4.1$, undergoes a period-doubling bifurcation (Fig. 7d). The attractor remains as a double-period loop (Fig. 7e, f) until $\gamma _{12}\approx 5.77$ where it doubles again (Fig. 7g, h). Such a sequence of period doubling is a hallmark of a chaotic system. Indeed, as $\gamma _{12}$ is increased further, the patterns cease to have obvious period patterns (Fig. 7i) and gain a rather more irregular look, suggestive of chaos.

## Discussion

We have used a class of diffusion–taxis systems for analysing the effect of between-population movement responses on spatial distributions of these populations. Our models are sufficient for incorporating taxis effects due to both direct and indirect animal interactions, so are of general use for a wide range of ecological communities. We have shown that spatial patterns in species distributions can emerge spontaneously as a result of these interactions. What is more, these patterns may not be fixed in time, but could be in constant flux. This brings into question the implicit assumption behind many species distribution models that the spatial distribution of a species in a fixed environment is roughly stationary over time.

Mathematically, our approach builds upon recent diffusion–taxis models of territory formation (Potts and Lewis 2016a, b). However, these latter models only consider two populations, and only in the case where there is mutual avoidance (i.e. Fig. 2c, d). We have shown that, when there is just one more population in the mix ($N=3$), the possible patterns that emerge can be extremely rich, incorporating stationary patterns, periodic oscillations, and irregular patterns that may be chaotic. Although irregular and chaotic spatio-temporal patterns have been observed in spatial predator–prey systems (Sherratt et al. 1995, 1997), this is one of the few times they have been discovered as arising from inter-population avoidance models (but see White et al. 1996, Section 8.2). These possibilities will extend to the situation of $N>3$, which is typical of most real-life ecosystems (e.g. Vanak et al. 2013).

The models studied here are closely related to aggregation models, which are well studied, often with applications to cell biology in mind (Alt 1985; Mogilner and Edelstein-Keshet 1999; Topaz et al. 2006; Painter et al. 2015). In these models, populations exhibit self-attraction alongside diffusion and are usually framed with just a single population in mind [although some incorporate more, e.g. Painter et al. (2015), Burger et al. (2018)]. In contrast to our situation, this self-attraction process can enable spontaneous aggregation to occur in a single population. Similar to our situation, in these self-attraction models it is typical to observe ill-posed problems unless some form of regularisation is in place, either through non-local terms (Mogilner and Edelstein-Keshet 1999; Briscoe et al. 2002; Topaz et al. 2006) or other means such as mixed spatio-temporal derivatives (Padrón 1998).

We have decided not to incorporate self-attraction into our framework. This is both for simplicity of analysis and because the animal populations we have in mind will tend to spread on the landscape in the absence of interactions, so are well described using diffusion as a base model (Okubo and Levin 2013; Lewis et al. 2016). However, in principle it is a simple extension to incorporate self-interaction into out framework, simply by dropping the $j\ne i$ restriction in Eq. (12). Indeed, for $N=2$, very similar models have been studied for aggregation/segregation properties (Burger et al. 2018) and pattern formation (Painter et al. 2015). In those studies, a combination of self-attraction and pursue-and-avoid can, contrary to the pure pursue-and-avoid case studied here, lead to moving spatial patterns where one aggregated population (the avoiders) leads the other one (the pursuers) in a ‘chase’ across the landscape (Painter 2009), a phenomenon observed in certain cell populations (Theveneau et al. 2013). For $N>2$, however, we have shown that the story regarding spatial patterns can already be very rich and complicated without self-attraction, so understanding the effect of this extra complication would be a formidable exercise.

Another natural extension of our work, from a mathematical perspective, would be to add reaction terms (a.k.a. kinetics) into our model, accounting for deaths (e.g. due to predation or as a result of competition) and births, by adding a function $f_i(u_1,\dots ,u_N)$ to Eq. (12) for each *i*. Biologically, this would change the timescale over which our model is valid, since in the present study we have explicitly set out to model timescales over which where births and deaths are negligible. Nonetheless, this extension is worthy of discussion since the addition of such terms leads to a class of so-called cross-diffusion models, which are well studied (Shigesada et al. 1979; Gambino et al. 2009; Shi et al. 2011; Tania et al. 2012; Potts and Petrovskii 2017). The term ‘cross-diffusion’ has been used in various guises, but the general form can incorporate both taxis terms of the type described here, as well as other terms that model various movement responses between populations. These cross-diffusion terms can combine with the reaction terms to drive pattern formation (Shi et al. 2011; Tania et al. 2012), as well as altering spreading speeds (Gambino et al. 2009; Girardin and Nadin 2015), and the outcome of competitive dynamics (Potts and Petrovskii 2017). The key difference between our work and traditional studies of cross-diffusion is that rich patterns form in our model despite the lack of kinetics. As such, we separate out the effect of taxis on pattern formation from any interaction with the reaction terms.

Our mathematical insights suggest that there is an urgent need to understand the extent to which the underlying movement processes in our model are prevalent in empirical ecosystems. Much effort is spent in understanding species distributions (Manly et al. 2002; Araujo and Guisan 2006; Jiménez-Valverde et al. 2008), often motivated by highly applied questions such as understanding the effect of climate change on biodiversity loss (Gotelli and Stanton-Geddes 2015), planning conservation efforts (Rodríguez et al. 2007; Evans et al. 2015), and mitigating negative effects of disease spread (Fatima et al. 2016) and biological invasions (Mainali et al. 2015). Species distribution models typically seek to link the distribution of species with environmental covariates, whereas the effect of between-population movement responses is essentially ignored. Presumably, this is because it is considered as ‘noise’ that likely averages out over time. In contrast, this study suggests that the patterns emerging from between-population movements may be fundamental drivers of both transient and asymptotic species distributions.

Fortuitously, recent years have seen the development of techniques for measuring the effects of foreign populations on animal movement. Animal biologging technology has become increasingly smaller, cheaper, and able to gather data at much higher frequencies than ever before (Wilmers et al. 2015; Williams et al., in review). Furthermore, statistical techniques have become increasingly refined to uncover the behavioural mechanisms behind animals’ movement paths (Albertsen et al. 2015; Avgar et al. 2016; Michelot et al. 2016; Potts et al. 2018). In particular, these include inferring interactions between wild animals, both direct (Vanak et al. 2013) and mediated by environmental markers (Latombe et al. 2014; Potts et al. 2014).

Consequently, the community of movement ecologists is in a prime position to measure between-population movement responses and seek to understand the prevalence of movement-induced spatial distribution patterns reported here. Our hope is that the theoretical results presented here will serve as a motivating study for understanding the effect of between-population movement responses on spatial population dynamics in empirical systems, as well as highlighting the need for such studies if we are to understand accurately the drivers behind observed species distributions.

## Electronic supplementary material

Below is the link to the electronic supplementary material.

Supplementary material 1 (mp4 1390 KB)

Supplementary material 2 (mp4 4821 KB)
